# New Perspectives in Third Molar Auto-Transplantation: Literature Review and a Case Report of Clinical, Financial and Forensic Implications

**DOI:** 10.3390/medicina60030473

**Published:** 2024-03-13

**Authors:** Alessandra Putrino, Enrico Marinelli, Alessandro Agrillo, Simona Zaami

**Affiliations:** 1 Department of Anatomical, Histological, Forensic and Orthopedic Sciences, Sapienza University of Rome, 00161 Rome, Italy; alessandra.putrino@gmail.com; 2 Department of Medico-Surgical Sciences and Biotechnologies, Sapienza University of Rome, 04100 Latina, Italy; enrico.marinelli@uniroma1.it; 3 Maxillofacial Surgery Unit, San Filippo Neri Hospital, 00135 Rome, Italy; alessandro.agrillo@tiscali.it

**Keywords:** oral surgery, auto-transplantation, third molars, clinical aspects in forensic implications

## Abstract

Third molar extraction is the most common procedure in oral and maxillofacial surgery. Third molars are considered less functional than other teeth and are often extracted. Sometimes, they are also used for auto-transplantation for the benefit of oral rehabilitation. Since many biological factors are involved in this surgical approach, herein, we outline a review of the biological characteristics of medico-legal/forensic interest, in addition to presenting a successful clinical case. A scoping review of currently available research data (following the principles of PRISMA-ScR or the Preferred Reporting Items for Systematic Reviews and Meta-Analyses extension for Scoping Reviews) on third molar auto-transplantation was conducted by drawing upon the main databases (Scopus, PubMed, Google Scholar and LILACS) to evaluate biological and clinical characteristics possibly relatable to forensic issues. All the collected data were summarized and elaborated on for the purpose of this article. A patient underwent extraction of the right upper first molar and auto-transplantation of the unerupted ipsilateral third molar. Many biologic and clinical factors are involved in the success of this clinical procedure. Knowledge of third molar anatomy, of its development and viable surgical approaches are all essential elements; just as important are the treatment of the tooth before and after transplantation and the integrity of the periodontal ligament. Follow-up of the clinical case for 5 years made it possible to verify the stability of the procedure over time. Third molar auto-transplantation is feasible and cost-effective. However, the use of third molars as donor teeth in auto-transplantation may have medico-legal implications. The lack of official protocols and consistent evidence-based guidelines for operators still prevent such a procedure from becoming mainstream; therefore, it is viewed with suspicion by clinicians and patients, even though the biological factors herein detected point to a reasonably high degree of safety. The understanding of many specific biological and clinical factors involved in the stability of third molar auto-transplantation allows for a thorough understanding of the forensic implications relevant to clinical practice. Effective communication and information provision are therefore of utmost importance, in the interest of both patients and doctors.

## 1. Introduction

Tooth loss is a sensitive indicator of overall dental health and the need for dental care. Since tooth loss is preventable, improvements in oral health education and improved quality of life, including dental care habits, have helped to mitigate such a phenomenon since the 1970s [1]. In spite of this improvement, significant disparities remain in some population groups. Financially disadvantaged people, those with low education levels and smokers are more than three times more likely to have lost teeth than the general population [2]. Tooth loss impacts the ability to maintain a healthy diet and can cause adverse psychological and social consequences [3,4]. This is extremely important in growing subjects, namely children and adolescents, for whom teeth play a key role in facial skeletal development [5,6]. Dentists routinely provide tooth loss rehabilitation with removable or fixed dentures, crowns or bridges and dental implants. Patients who have not yet completed skeletal development, like children and young patients, can be rehabilitated with removable prostheses only [7]. This encourages bone loss in edentulous areas and may complicate future rehabilitation [8], in addition to negatively affecting quality of life [9]. Another rehabilitative strategy, maybe less known by patients and less proposed and used by dentists, is dental auto-transplantation, which is the surgical transposition of a tooth from its original site to another site to replace a lost or compromised tooth in the same individual [10]. Tooth auto-transplantation has been described in the literature as a valid choice in growing subjects because these patients would not yet be ready to receive implant rehabilitation and can benefit from an economic and aesthetic treatment [11]. There is no risk of losing alveolar bone or space, and natural proprioception is preserved [12,13]. The third molars, despite their high levels of anatomical variability and complexity, are widely used in this procedure, as they are posterior teeth with high risk of disodontiasis or inclusion that can produce inflammation or caries in nearby dental elements [14]. In the mandible, extractive surgery of third molars for reasons other than auto-transplantation can play an important role in ambulatory dentistry, even if their position and angle of development may be positively affected by the extraction of the first molar [15,16]. Furthermore, third molar surgery has evolved over time, and current approaches mostly aim to reduce post-operative sequelae (pain, facial swelling and trismus) by improving patient comfort [17]. Even when they are healthy, third molars are viewed as “expendable”, since they are considered less functional than other teeth and their prophylactic removal is judged to be the more cost-effective strategy [18,19]. In growing patients, transplanted third molars help maintain the ability to continue maturation as if they were in their original location, while in adults or young subjects in whom root maturation is complete and the apices are closed, endodontic and prosthetic rehabilitation of the transplanted third molar is needed in order to preserve tooth stability and improve the prognosis over time [20,21]. The survival rate and success do not appear to be affected by transplantation to fresh extraction sites (for immediate auto-transplantation) or to surgically prepared sites (for delayed transplantation) with or without guided bone regeneration (GBR) [12]. The success of the procedure seems to be connected to an appropriate execution by the operator but also as well as to the ability to carefully select the patient. However, unclear biological factors affect the long-term result of third molar auto-transplantation both in growing patients and in adults [13,18]. Given the current lack of conclusive data on auto-transplantation of third molars as an alternative strategy to more traditional rehabilitation approaches, such a procedure remains limited in its scope of application and only usable by relatively few, highly experienced clinicians [18,20,21,22,23]. From a medico-legal standpoint, dentists have a duty to guarantee the best and most predictable result [24]. Given the absence of decisive research data and evidence-based guidelines, thorough and effective communication as to the risks associated with dental, rehabilitative, surgical and non-surgical procedures is even more crucial [24,25]. Dental surgical practice is of particular interest because it encompasses both the smile’s esthetic value and the masticatory function [24]. According to the latest WHO Global Oral Health Status Report (GOHSR), millions of people of all ages worldwide suffer from incomplete dentition due to the premature loss of dental elements as a consequence of untreated tooth decay or periodontitis [26]. This procedure, when safely and properly used, could have a relevant economic and social impact. This scoping review is centered around the possible biological and clinical factors and mechanisms related to third molar auto-transplantation that may have forensic implications in order to support clinicians and patients; the case report herein presented lays out a clinical case with 5-year follow-up of a young adult patient with an unerupted third molar auto-transplanted to replace a compromised first molar in the upper arch. 

## 2. Materials and Methods

### 2.1. Scoping Review

The search of sources useful for the scoping review was finalized on 5 November 2023 by adhering to the principles of PRISMA-ScR (Preferred Reporting Items for Systematic reviews and Meta-Analyses extension for Scoping Reviews) [27]. Two independent operators were tasked with delving into the Scopus, PubMed, LILACS and Google Scholar scientific databases. The research question was “Are biological and clinical mechanisms and factors with forensic implications in third molar auto-transplantation used in adult and growing patients described in the literature?”. Such a query was devised according to the acronym PCC (population/problem, concept, context), as briefly laid out in Table 1. The following MeSH terms and free terms were used as search strings in combination with the Boolean operators “AND” and “OR”: tooth auto-transplantation, third molars, wisdom teeth, oral surgery, biologic factors, legislation and jurisprudence. The eligibility criteria were established as follows: English language, availability of abstracts and full-text versions and randomized and non-randomized clinical studies on humans. Reviews, case reports/series, in vitro/in vivo studies on animals and research published in other languages or not related to the aim of the review were excluded (inclusion and exclusion criteria have been outlined in Table 2). No time restrictions have been set. The review was carried out autonomously by two experienced operators, namely a dental researcher and a university professor in forensic medicine, both experts in their field and with experiences in each other’s specialty. The results found in the consulted databases were initially unified; then, duplicates were manually removed. Reading of the abstracts and the subsequent full-text reading allowed the reviewers to obtain the final number of useful sources. Data were always extracted independently and in duplicate, then compared and unified. Doubts were dispelled and clarifications provided through the supervision of a third operator experienced in forensic medicine with an active academic position. For each study, the following information was extracted: authors, year, study design, number of patients, age and sex (if described), third molar stage of maturation, biological mechanisms and clinical factors with potential forensic implications. 

### 2.2. Case Report

**Figure 1:**
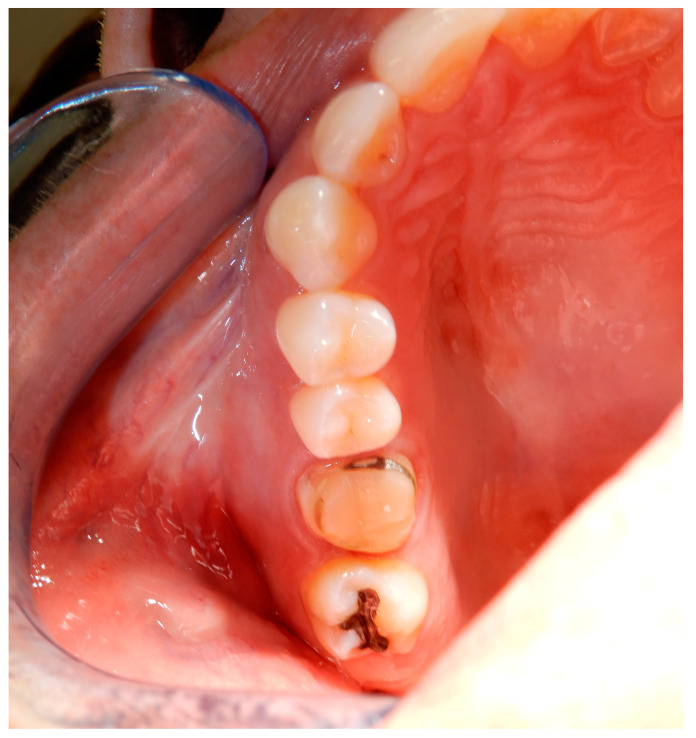
Occlusal view of the right side of the upper dental arch. There are no obvious clinical signs related to the pain symptoms reported by the patient. The prosthetic crown on the first molar was removed.

**Figure 2:**
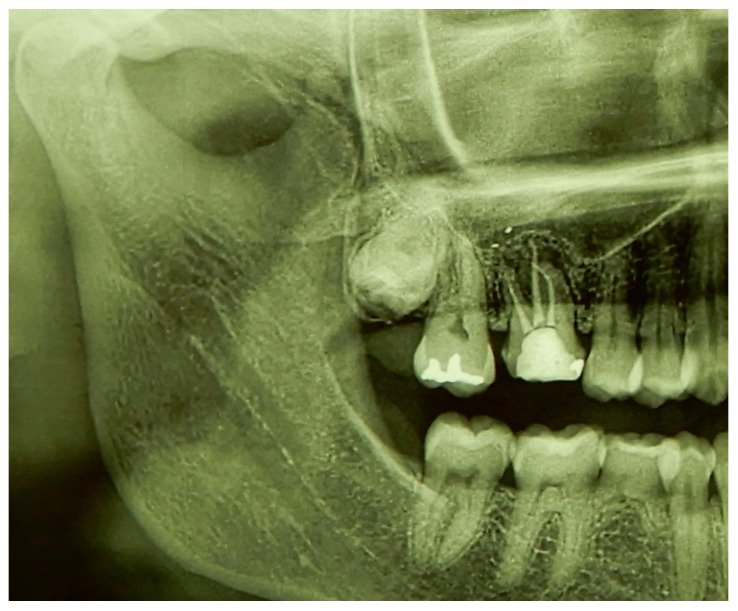
Detail of the dental radiograph showing the pre-existing endodontic treatment of the upper-right first molar with periapical inflammation and the presence of the third molar included in the bone.

A female young adult patient (35 years old) came to the dental office complaining of widespread pain in the posterior sector of the upper dental arch. This persistent pain caused her a strong functional limitation in terms of both chewing and simple contact between the dental arches when at rest. Given the absence of clinical signs in the area affected by the symptoms (Figure 1), a dental radiograph was requested. The orthopanoramic radiograph of the dental arches revealed the presence of a periapical lesion on the first upper-right molar, which had already been treated endodontically and covered by a prosthetic crown (Figure 2). Furthermore, the third molar on the same side appeared impacted with the root apices closed (Figure 2). The therapeutic strategy involved the extraction of the first molar and, at the same time, the extraction and recovery of the third molar as a replacement dental element in place of the first molar, both of which were proposed to the patient. The presence of closed apices in the third molar, as explained to the patient, would require the endodontical treatment of the tooth following its insertion into the recipient site and its protection by means of prosthetic crown coverage. Despite the possibility of failure, the biological and economic advantage of this auto-transplantation was more reasonable for the patient compared to alternatives such as endodontic retreatment of the first molar, third molar extraction or extracting both elements and rehabilitation of the first molar edentulous area via prosthetic endosseous implant. 

## 3. Results

### 3.1. Scoping Review

**Figure 3:**
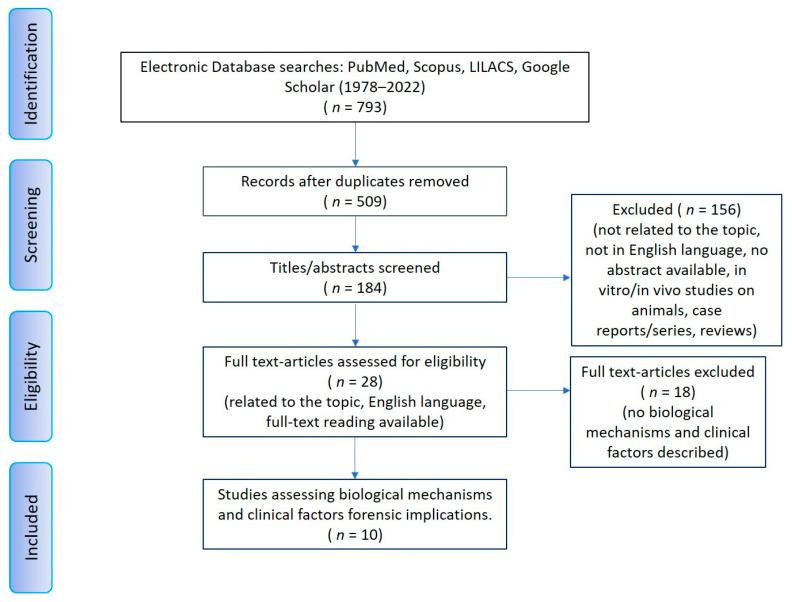
The PRISMA flow diagram for the systematic review detailing the database searches, the number of abstracts screened and the full texts retrieved.

The database search initially yielded 793 results (PubMed, Scopus, Cochrane Library and LILACS) published between 1978 and 2023. Duplicates were manually removed. The remaining 184 results were checked for eligibility criteria by reading the abstracts. Following this step, 156 articles were excluded, since they did not meet the established eligibility criteria. Out of the remaining 28 articles that were assessed as eligible for full-text reading, 18 were excluded, since they reported no biological factors or mechanisms involved in third molar auto-transplantation. The ten articles left were included in the scoping review, since they assessed biological mechanisms and clinical factors with potential forensic implications (Table 3). From the supplemental research of the scientific sources herein included and cited, no further suitable studies were found (Figure 3). The scoping review relied on sources published over the past two decades, from 2002 to 2022. The total number of patients involved in the studies documenting the use of third molar auto-transplantation ultimately totaled 573 [28,29,30,31,32,33,34,35,36,37]. The distribution between the two sexes was not available; hence, no estimation based on this variable could be made as to the incidence of biological factors or mechanisms. Patients were in the 15–53 age range. Only three studies did not provide this information [31,32,37]. As for the study types, five out of ten studies were retrospective [28,30,32,34,36], two studies were observational [35,37], one study was comparative [31], one was defined by its authors as prospective [33] and one was registered as a clinical trial [29]. All but two studies [28,32] clarified the stage of formation of the third molars used for auto-transplantations. Three studies used third molars with an immature root formation stage [29,31,35], three other studies referred to the use of third molars with fully mature roots [30,36,37] and, finally, two other studies documented the use of both levels of root formation [33,34]. The most commonly used classification to define the stage of root maturation was Moorrees classification [29,31,34,35,38]. Biological mechanisms and clinical factors, may have important forensic implications, turned out to be varied, including the timing of healing and bone formation, including transient mobility [28,29] and stimulation at bone deposition in deficient sites [35]; the effect of the forces expressed by adjacent teeth [30]; the root response according to the stage of root maturation [31,33]; the influence by the recipient bone according to its characteristics to be evaluated radiographically in growing subjects [32]; and the role of the periodontal ligament in surgery and over time [34,36,37].

### 3.2. Case Report

**Figure 4:**
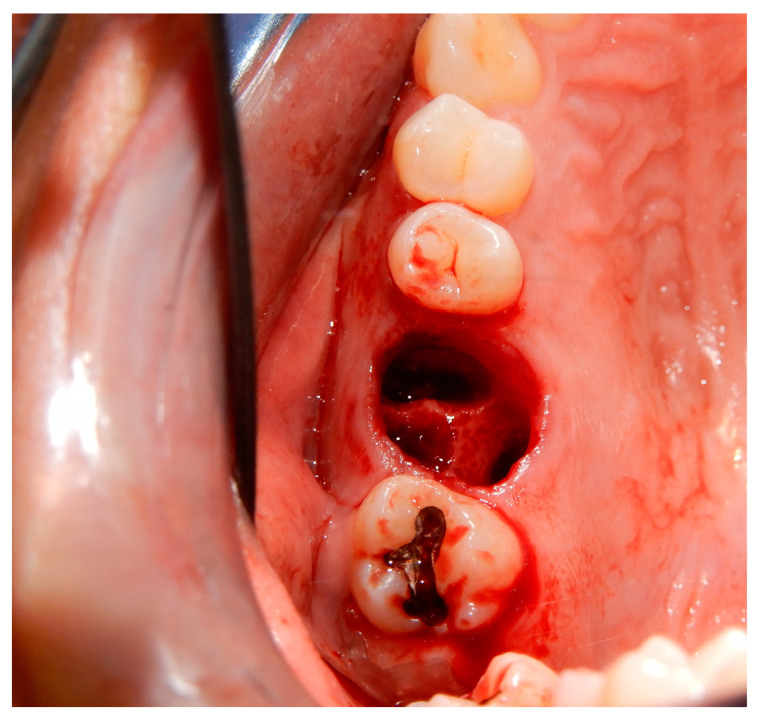
Post-extraction empty socket (of the right upper first molar) that will serve as the recipient site (of the right upper third molar).

**Figure 5:**
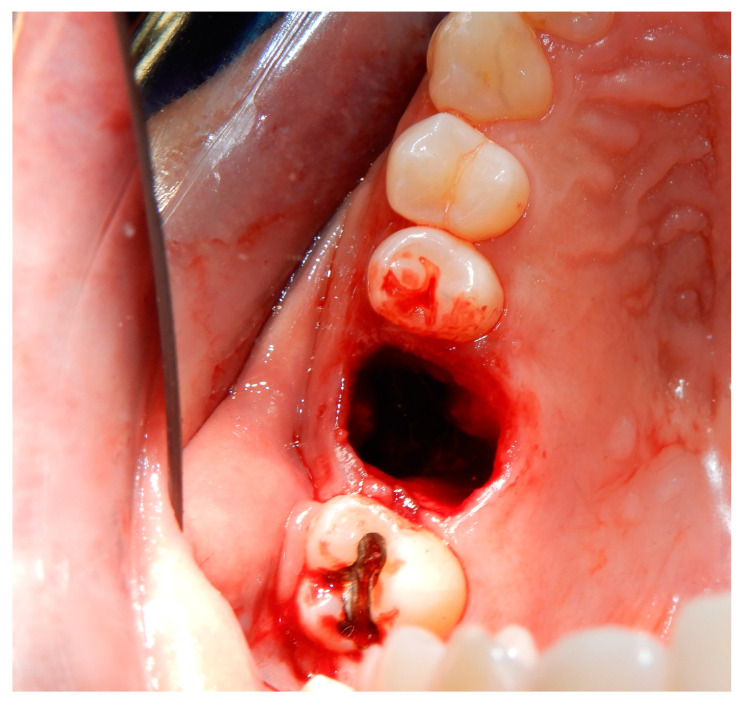
Post-extraction empty socket (of the upper-right first molar) from which the interradicular septum was removed to position and fit with the donor tooth (third molar).

**Figure 6:**
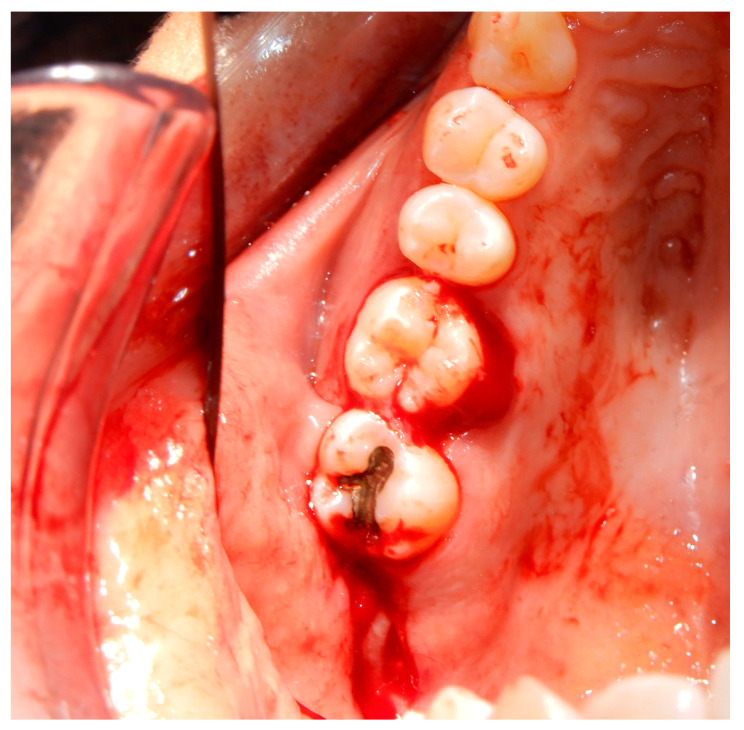
Transplanted third molar inserted into the recipient site with mild infra-occlusion. Distal to the second molar, the linear incision of extraction of the transplanted third molar is visible.

**Figure 7:**
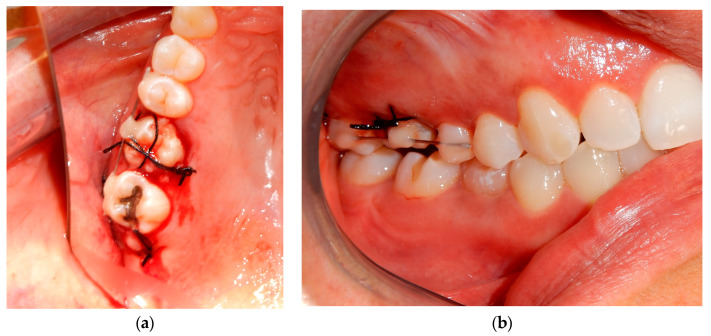
Non-absorbable silk suture and orthodontic splinting. (**a**) In the occlusal view, both the suture at the extraction site of the third molar and the cross-bridging suture on the transplanted third molar are visible. (**b**) In the buccal view of orthodontic splinting and the suture, the deliberately infraoccluded position of the transplanted third molar is appreciable.

**Figure 8:**
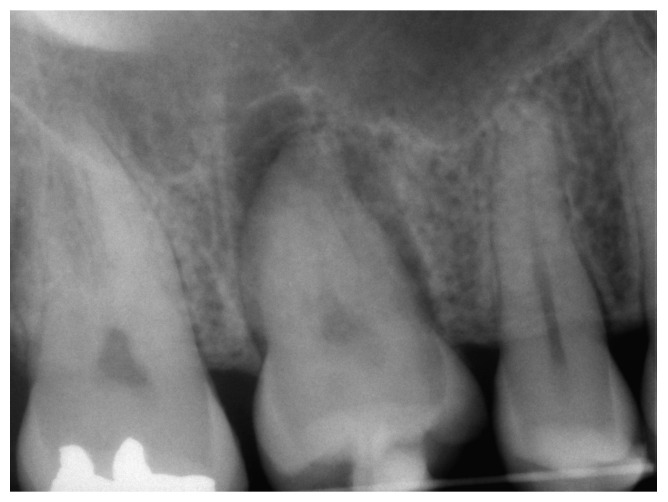
Post-operative periapical X-ray.

**Figure 9:**
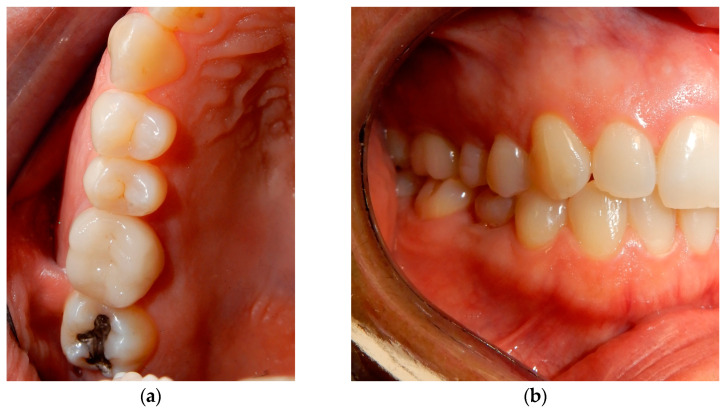
Zirconia Prosthetic crown rehabilitation. (**a**) Occlusal and (**b**) buccal views.

**Figure 10:**
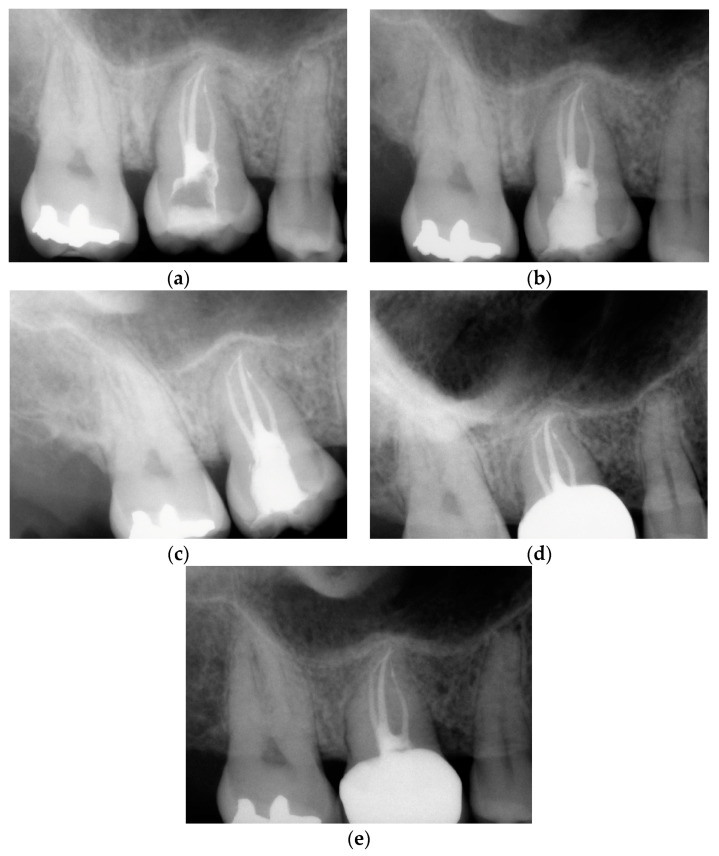
Periapical control radiographs. (**a**) After 3 months, endodontic therapy was performed; (**b**) after 6 months; (**c**) after 12 months; (**d**) after 18 months, prosthetic coverage with a dental crown in zirconia was applied; (**e**) after 36 months.

**Figure 11:**
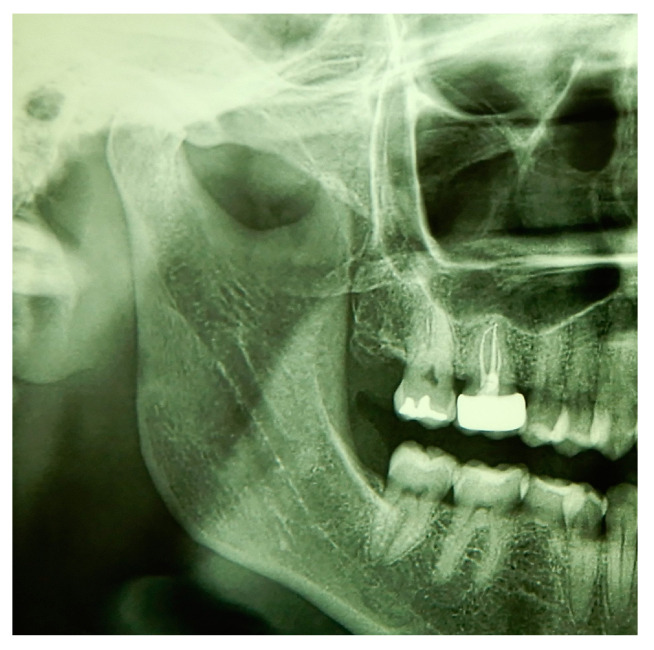
Radiographic control with orthopanoramic X-ray 5 years after auto-transplantation of the third molar.

**Figure 12:**
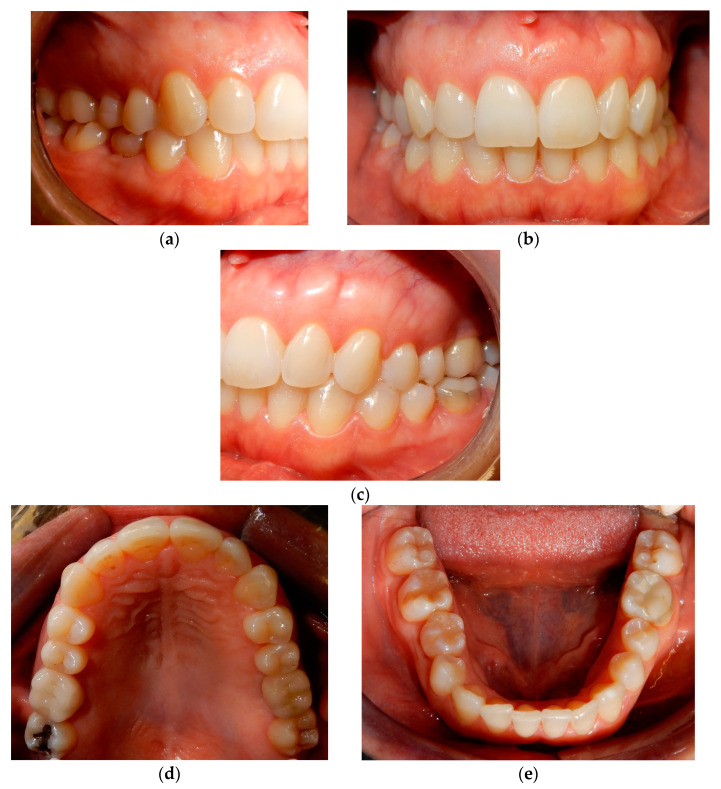
Final case photos. (**a**) Right side view; (**b**) frontal view; (**c**) left side view; (**d**) upper occlusal view; (**e**) lower occlusal view.

Before starting the various surgical and rehabilitative phases for third molar auto-transplantation at the site of the compromised first molar, the patient was informed of the risks related to treatment failure, the lack of conclusive guidelines and the possible need to resort to traditional rehabilitation options if the auto-transplantation did fail. Knowledge of currently available research findings was shared with the patient in the form of oral communication comprehensible to a person with no professional expertise in dentistry. A personalized, specifically tailored informed consent for the case to be treated with the aforementioned auto-transplantation procedure was drafted, then read, discussed and signed by both the clinician in charge of the treatment and the patient. Auto-transplantation of the third molar in place of the first molar was performed in a single surgical session. The operational steps unfolded as follows: extraction of the first upper molar, extraction of the third molar included in the bone, insertion of the third molar into the empty alveolus of the first molar, placement of sutures in both sites and orthodontic splinting held for 2 weeks. Endodontic therapy was performed 3 months after transplantation; a protective crown was added a year and a half later. After checking that the teeth were clean, the first step, after the use of mouthwash with chlorhexidine 0.12% for 1 min, was the extraction of the compromised first molar, which was routinely performed after infiltrative loco-regional anesthesia. The avulsion was performed using the combination of forceps and a lever. The bottom of the empty socket was gently cleaned with an endoalveolar spoon, and the interradicular septum was provisionally left (Figure 4). After a few minutes, again under infiltrative local anesthesia, a distal flap was opened to the second molar to extract the included third molar with a surgical lever. The latter presented with a single root trunk and was kept in sterile saline solution for 5 min, during which time the residual interradicular septum was removed from the site of the first molar (Figure 5) and immediately inserted slightly infraoccluded into the recipient site (Figure 6). Once the surgical site of extraction was sutured with a non-absorbable silk suture thread, the implanted element was then secured both with the same type of thread, applied as a cross bridge on the occlusal side and with a splinting from the buccal side made with 0.12 steel orthodontic wire and composite resin. It was extended from the second premolar to the second molar (Figure 7). The patient underwent pre- and post-surgical antibiotic coverage for a total of 5 days (starting from the evening before surgery) based on amoxicillin and clavulanic acid (875 mg/125 mg two times a day) and non-steroidal anti-inflammatory medication as needed for any post-operative pain. She was advised to perform normal oral hygiene on all teeth except the splinted teeth but to clean the surgical sites with a cotton swab soaked in hydrogen peroxide until the suture was removed. The splint was kept in situ for two weeks. Endodontic therapy with endocanal filling was performed after 3 months (System B™ Endodontic Heat Source, Kerr Endodontics, Gilbert, AZ, USA) (Figure 8). In the absence of symptoms, the tooth was covered with a zirconia crown after 18 months (Figure 9). The patient underwent periodic checks, some of which included periapical radiographs (3 months, 6 months, 12 months and 18 months after the surgical procedure) (Figure 10a–e) or in conjunction with professional hygiene sessions to verify the absence of mobility and the presence of normal periodontal health indices (Gingival Index, Bleeding on Probing, PPD and CAL). The SARS-CoV-2 pandemic delayed the control after that of 3 years. A radiographic and clinical check-up 5 years after the auto-transplantation surgery documents the perfect state of health of the transplanted tooth (Figure 11 and Figure 12).

## 4. Discussion

Dental auto-transplantation refers to the transplantation of a tooth from its original site to another in the same human mouth [10,39]. The donor tooth can be erupted or impacted. The recipient site can be a surgically prepared socket in the case of a missing tooth due to congenital reasons or old extractions or a fresh, empty socket when a non-restorable tooth, usually a first or second molar, has been extracted [11,40]. Third molars are the most common donor teeth used in this procedure [12,18,20,21,22,23]. In fact, third molars are often extracted for various reasons, including difficult or impossible eruption, frequent pericoronitis or impingement of the adjacent lower second molar [41]. The use of premolars, canines and supernumerary teeth is also documented in literature [10,13,42,43,44,45]. From a practical and medico-legal point of view, it must be considered that being a maxillary or mandibular donor tooth or recipient site does not affect the success of the auto-transplantation of third molars and of other permanent teeth [34]. The reported success rates of third molar auto-transplantation are around 94% in donor teeth with incompletely formed roots and 89% for donor teeth with completely formed roots [10,11,34,35,39,40]. However, the immediate and long-term prognosis of auto-transplanted permanent teeth is influenced by well-established and clear primary factors related the selection criteria, the extraoral management of auto-transplanted teeth, the fixation techniques and their duration and the choice to use or not use endodontic treatment of the auto-transplanted tooth based on its root maturation stage [10,11,13]. The biological factors and mechanisms with medico-legal implications commonly described in the studies included in this review are represented by the stage of root maturation of the third molar and periodontal ligament preservation [28,29,30,31,32,33,34,35,36,37]. Most studies refer to the Moorrees classification [38] and encourage the safe use of mature third molars much more than in the past [30,33,36,37], expanding the possibilities of acting with success not only on young or growing subjects but on adult patients as well [33]. The 5-year follow-up of our clinical case confirms the safe use of mature third molars in auto-transplantation and the principle that the root development stage influences only the therapeutic approach but not the result. The presence of open apices allows for pulp revascularization and helps to preserve proprioception [33]. This is certainly an advantage compared to transplanted teeth with an already closed apex and, consequently, with complete root maturation. However, the use of mature third molars is not a contraindication and can therefore be offered as a treatment to adult patients without the risk of incurring any negligence or inexperience except in the case of incorrect evaluation and management of the case. Furthermore, after bone healing transplanted third molars, can be moved orthodontically, although it is important to know about the auto-transplantation before any application of orthodontic forces [29,32]. However, it is not yet clear whether there is a time beyond which orthodontic movement on a third molar or on another auto-transplanted tooth can be considered safer. The answer to this question also needs to be evidence-based in order to support the clinician or the patient in the case of medical malpractice litigation. An important fact from a biological and medico-legal standpoint is that orthodontic extrusion and minor lateral movements of auto-transplanted immature third molars, as well as rotation of single-rooted third molar transplants, represent no additional risk to transplant survival. When the rotation occurs in a multi-rooted transplanted tooth, it seems to initiate later severance of the vascular and nerval supply to the pulp [29]. This could be in accordance with our experience, since in our clinical case, the third molar was inserted without any rotation, even though its root shape was simple enough. If we consider the possibility of orthodontically closing the edentulous spaces of the first molars by mesialization and/or distalization of the adjacent teeth, although these movements can also be performed with clear aligners, they are not free from risks for the teeth on which the body movement takes place [45]. Functional aspects are just as important. In fact, some research findings show that proximal grinding may be associated with failure in open apices of third molars [33]; however, when the teeth adjacent to the transplanted molar exert indirect friction on it, it remains stable even if mature [29]. In our experience, the transplanted molar was only inserted slightly infra-occluded, then released from the occlusion with the antagonist tooth to avoid harmful stresses from chewing or clenching. Another factor with forensic implications is that related the mobility of the transplanted tooth because within 3 months, it corresponds to normal bone formation. Bone healing at the recipient sites completely occurs within 6 months with the appearance of lamina dura [28]. This timing was also radiographically detected in our clinical case. According to the same group of authors, endodontic therapy on third molars should not have an influence on bone healing, so it would seem that the choice of immediate endodontic therapy or postponement for weeks or months has no medico-legal repercussions [28]. Our choice to proceed with 3-month deferred endodontic therapy was linked to better healing of the peri-implant site and the possibility of isolating the tooth more easily with a rubber dam in the absence of splinting. With regard to orthodontic splinting, in the selected literature, there does not seem to be a single orientation with respect to the type of method to be used nor with respect to the time of application on the third molars. Therefore, we maintained the methods and duration of application used for the other types of auto-transplanted teeth [10,13,42,43,44,45,46]. Still, the bone response visible on radiographs confirms the safety of auto-transplantation, for example, in the successful emergency treatment of complications of an oro-antral fistula [32,47]. It allows for the extension of auto-transplantation of third molars beyond therapy for a permanent edentulous site. Bone response evaluation for clinical and medico-legal implications is also important in the case of rehabilitation of an agenesic site, when the excessive growth of the alveolar process is expected based on cephalometric analysis, since in the auto-transplanted tooth, ankylosis is more likely [35]. However, as far as dental auto-transplantation research goes, no studies seem to address whether the quality of the initial bone of the recipient site can affect procedural outcomes. Recent evidence points to bone grafts in atrophic alveolar areas as a successful option in the pre-auto-transplantation phase, in addition to auto-transplantation itself, which is indicated in the management of deficient alveolar ridges [48,49]. More generally, remodeling processes (formation and resorption) permanently occur in dental bone tissue and are modulated by bone metabolism [50]. Taking into account the fact that bone is one of the main targets of hormones and endocrine diseases, its preoperative quality should be assessed when auto-transplantation is considered as a therapeutic option in patients affected by endocrine problems or under pharmacological therapy with hormones [51]. Furthermore, human osteoblasts exhibit sexual dimorphism, which suggests a different substrate-dependent response [52]. The integrity of the periodontal ligament is another crucial point. If properly preserved on the third molar, as well as on the recipient site, prognosis and periodontal health will improve, especially if Hertwig’s epithelial root sheath is not damaged [31,34,36,37]. This could be due to the potential of Hertwig’s epithelial root sheath cells for periodontal tissue regeneration [53]. Third molars at advanced stages of development show a lower post-operative root development, probably due to damage to Hertwig’s epithelial root sheath during the transplantation procedure [31]. For this reason, the maintenance of the tooth in saline solution should not exceed a critical time of 18 min [32]. In our experience, a careful organization of the team undertaking the procedure and a proper preoperative evaluation can significantly reduce this time, which, in our case, did not exceed 5 min. Some authors claim that the use of a replica element of the donor can help to reduce the extraoral time of the tooth to be transplanted because it allows the recipient site to be adapted more precisely [54]. In our experience, the phase of removal of the interradicular septum of the recipient site did not present any major issues, partly thanks to the simple root morphology of the third molar. Monitoring periodontal parameters in the first 6–12 months allows for consideration of safe auto-transplantation in the absence of periodontal inflammation and when the depth of probing is less than 4 mm after the first year, even in the presence of complete root development in the third molar [36,37]. In our experience, the periodical monitoring of periodontal healing during professional hygiene sessions enabled us to guide the prosthetic timing as well, which can be very important from a medico-legal standpoint in the event of litigation. According to the evaluations discussed by other authors, the contraindications for auto-transplantation include mainly the absence of an adequate tooth for auto-transplantation, the absence of space for rehabilitation, the absence of alveolar bone tissue in the recipient site, an inadequate stage of development of the tooth to be auto-transplanted and the poor general and oral health status of the patient [55]. The main limitation of our case has to do with the fact that, having been considered a simple case due to the anatomy of the third molar, the orthopantomography of the dental arches and the intraoral periapical control radiographs were used as initial and control radiographic references, respectively. Although the need for a second-level diagnostic imaging examination (e.g., a CT-Dentascan) has never been reported by any scientific source [28,29,30,31,32,33,34,35,36,37], we do believe that such an option may be valuable when the anatomy of the recipient/donor site and/or of the element to be transplanted are complex. The lack of specifically targeted evidence-based guidelines governing timing and procedures for the auto-transplantation of third molars (applicable to other teeth as well) means that a biologically valid procedure (and one with major economic and social impact as well), especially for younger patients, is not considered viable by many clinicians and risks being viewed with suspicion, even by patients who have undergone such a procedure [56]. In our case, the therapeutic pathway has been solidly grounded in a comprehensive information provision, aimed at clarifying the risks of failure and the need to restore oral function with traditional treatments (endosseous prosthetic implant of the extracted tooth). The process of building awareness, on which the decision-making process must be based, was certified by a specifically tailored informed consent, in writing, as a cornerstone and fundamental safeguard for all the parties involved [57]. In that respect, it is worth stressing that the issues relating to the information and consent acquisition phase are part and parcel of the conceptual evolution that defines the qualitative aspects of care. Such key elements are also instrumental in the timely planning of therapeutic protocols [58] and must always be guided by diagnostic–therapeutic “personalization” to prioritize results and sustainability. As for the forensic value of the radiographic finding of an auto-transplanted tooth in personal identification, it is still a rather under-researched aspect for which more conclusive research data are needed. In fact, a dental profile based on traditional dental impressions, or on the most frequent intraoral scans may fail to highlight such an individual trait. However, computerized matching of common radiographs with dental impressions or scans has been reported to be potentially helpful in personal identification and may possibly shed a light on the original root anatomy of the self-transplanted tooth [59]. Such a potential can make it an important biological factor that provides guidance for the identification and reconstruction of a living or deceased subject. In the attempt to propose a general draft of a protocol for third molar auto-transplantation and based on our review results, we recommend a thorough assessment of oral and general factors prior to third molar auto-transplantation. Orthopantomography of the teeth is a sufficient initial examination if the anatomy of the donor tooth and the recipient site are not complex, but an examination such as CBCT can be helpful if there are anatomical complexities and/or if the case requires the design of a replica donor tooth. Oral systemic antibiotics should be administered a few hours before the procedure and taken for 5 days. Teeth should be cleaned, and surgical sites should be disinfected. The donor and recipient sites should receive anesthetics at the same time. The surgical procedure should be atraumatically performed, avoiding any damage to the donor tooth and its periodontal ligament but also to the bone and empty socket in the recipient site. Forceps and levers may be safely used. The recipient site may be fresh or surgically created with the help of a donor tooth replica designed and printed with modern instruments. As soon as possible (less than 10 min), the donor tooth from the saline solution should be placed in the recipient site in slight infraposition, leaving it free from occlusal and articulation forces. After checking the stability of the auto-transplanted tooth, the surgical sites should be sutured, and post-operative fixation should be carried out with a suture crossing the occlusal surface for 7 days. The site should be splinted with a thin and flexible orthodontic wire for 2 weeks. Anti-inflammatory medication can be taken for any post-operative pain. If the auto transplanted third molar has closed apices, endodontic therapy with endocanal filling should be performed after 3 months and covered with a prosthetic crown after 18 months. If the third molar has open apices, natural maturation should occur. Periodic periapical radiographical checks should be planned (3 months, 6 months, 12 months and 18 months after surgery). At the same time, the absence of mobility and the presence of normal periodontal health indices (Gingival Index, Bleeding on Probing, PPD and CAL) should be clinically assessed.

## 5. Conclusions

Missing teeth can be safely replaced through third molar auto-transplantation. Several factors can affect the success rate of such a technique. Periodontal ligament preservation plays a key role. The subsequent steps of positioning, stabilization and endodontic treatment, if apices are closed, can decisively contribute to the procedure’s success in the long term.

The indisputable advantage of using third molars to replace one or more missing teeth may have substantial social and economic implications for adult patients. In this case, even if endodontic therapy and prosthetic coverage by an artificial dental crown are required, other more expensive prosthetic rehabilitations can be avoided. In growing subjects, when teeth still have open apices, the proprioception of the natural transplanted tooth can be maintained. Orthodontic forces can be safely applied, and the insertion of implant prostheses can be avoided or deferred if the self-transplanted tooth is lost over time. The lack of standardized protocols, however, can considerably hinder the mainstream application of this rehabilitation strategy by clinicians, opening the way for forensic and medico-legal discussions as to the real potential of such a technique to guarantee favorable results as opposed to other more well-established rehabilitation avenues. 

## Figures and Tables

**Table 1:** Research question based on the PCC (population/problem, concept, context) strategy.

Population/Context	Adult and Growing Patients
Concept	Biological mechanisms and/or clinical factors that may be involved in forensic implications
Context	Third molar auto-transplantation

**Table 2:** Inclusion and exclusion criteria.

Inclusion Criteria	Exclusion Criteria
Inherence with the topic	Non-inherence with the topic
English language	Other languages
Abstract and full-text reading available	No abstracts and/or full-text reading
Randomized and non-randomized clinical studies on humans	In vitro/in vivo studies on animals, case reports/series and reviews

**Table 3:** Studies reporting biological mechanisms and/or clinical factors with forensic implications in third molar auto-transplantation selected for the scoping review.

Authors/Year	Study Design	Sample Size	Third Molars	Biological Mechanisms and Clinical Factors
Waikakul A. et al., 2002 [28]	Retrospective Study	14 patients (28–53 years)	Stage of development not specified	Mobility within 3 months corresponded to normal bone formation. Bone healing at the recipient sites completely occured within 6 months (50% had lamina dura). There was no significant association between the EPT response and bone formation.
Bauss O. et al., 2004 [29]	Clinical Trial	88 patients (17.3 years)	Immature third molars (stage 3 or 4 of root development)	Orthodontic extrusion and minor lateral movements of auto-transplanted immature third molars, as well as rotation of single-rooted third molar transplants, represent no additional risk to transplant survival. In contrast, rotation of multi-rooted transplants seems to initiate later severance of the vascular and nerval supply to the pulp.
Akkocaoglu M. et al., 2005 [30]	Retrospective Study	78 patients (18–24 years)	Fully developed roots	When the teeth adjacent to the transplanted molar are able to exert indirect friction on it, it remains stable even if mature.
Bauss O. et al., 2008 [31]	Comparative Study	62 patients	Immature third molars (stage 3 or 4 of root development)	Third molars at advanced stages of development showed lower post-operative root development, probably due to damage to Hertwig’s epithelial root sheath during the transplantation procedure.
Bokelund M. et al., 2013 [32]	Retrospective Study	157 patients	Stage of development not specified	Radiographic controls allowed the authors to conclude that there is a high risk of ankylosis when a permanent tooth is transplanted in a recipient site where the primary tooth was in infraposition and permanently missing for agenesis.
Nagori SA et al., 2014 [33]	Prospective Study	57 patients (15–25 years)	Fully or partial developed roots	Open apices allow for pulp revascularization. Proximal grinding of donor teeth is associated with failure.
Tang H. et al., 2017 [34]	Retrospective Study	23 patients (29.6 years)	Fully or partially developed roots (stages 4 and 5 of root development)	Preservation of both the PDL at the recipient site and that attached to the transplanted tooth is essential. Physiological saline solution and extra oral time less than 18 min influence PDL vitality.
Assad M. et al., 2018 [35]	Observational Study	20 patients (20–40 years)	Immature third molars (stage 3 or 4 of root development)	Auto-transplanted third molars can provide bone deposition for sinus borders and closing of the oro-antral fistula.
Kamata Y. et al., 2021 [36]	Retrospective Study	14 patients (28–53 years)	Fully developed roots	Periodontal healing parameters (PPD, CAL and KGW) values after 6 and 12 months allow for consideration of safe auto-transplantation even in the presence of complete root development in the third molars.
Maddalone M. et al., 2022 [37]	Observational Study	60 patients	Fully developed roots	Outcomes are influenced by accurate care of the periodontal ligament after extraction, proper stabilization and a depth of probing less than 4 mm in the first year.

## Data Availability

Not applicable.
